# Supplementary material for: Therapeutic patient education programs on diabetes in sub-Saharan Africa: A systematic review
Source: PLoS One. 2024 Jun 27;19(6):e0299526. doi: 10.1371/journal.pone.0299526 (PMC11210798; doi:10.1371/journal.pone.0299526)
Supplement: S1 Appendix — (DOCX) [file pone.0299526.s001.docx]

**S1 Appendix : Search strategy**

| **Database** | **Equation** | **Items Identified** |
| --- | --- | --- |
| PubMed | ((diabetes[MeSH Terms]) AND ((((((("patient education"[Title/Abstract]) OR ("self-management"[Title/Abstract])) OR ("patient compliance"[Title/Abstract ])) OR ("therapeutic education"[Title/Abstract])) OR ("self-care"[Title/Abstract])) OR ("health education"[Title/Abstract])) OR ("education programme"[ Title/Abstract]))) AND ((((((((((((((((((((((((((((((((((((((( ((((((africa[MeSH Terms]) OR (angola[MeSH Terms])) OR (benin[MeSH Terms])) OR (botswana[MeSH Terms])) OR (burundi[MeSH Terms])) OR ( burkina faso[MeSH Terms])) OR (Cameroon[MeSH Terms])) OR (cape verde[MeSH Terms])) OR (central african republic[MeSH Terms])) OR (chad[MeSH Terms])) OR (comoros [MeSH Terms])) OR (congo[MeSH Terms])) OR (cote d'ivoire[MeSH Terms])) OR (djibouti[MeSH Terms])) OR (equatorial guinea[MeSH Terms])) OR (eritrea[MeSH Terms])) OR (ethiopia[MeSH Terms])) OR (gabon[MeSH Terms] ])) OR (gambia[MeSH Terms])) OR (ghana[MeSH Terms])) OR (guinea[MeSH Terms])) OR (guinea bissau[MeSH Terms])) OR (kenya[MeSH Terms])) OR (lesotho[MeSH Terms])) OR (liberia[MeSH Terms])) OR (madagascar[MeSH Terms])) OR (malawi[MeSH Terms])) OR (mali[MeSH Terms])) OR (mauritania[MeSH Terms] ])) OR (mauritius[MeSH Terms])) OR (mozambique[MeSH Terms])) OR (namibia[MeSH Terms])) OR (niger[MeSH Terms])) OR (Nigeria[MeSH Terms])) OR ( rwanda[MeSH Terms])) OR (sao tome and Principe[MeSH Terms])) OR (senegal[MeSH Terms])) OR (seychelles[MeSH Terms])) OR (sierra leone[MeSH Terms])) OR (somalia [MeSH Terms])) OR (south africa[MeSH Terms])) OR (sudan[MeSH Terms])) OR (swaziland[MeSH Terms])) OR(tanzania[MeSH Terms])) OR (togo[MeSH Terms])) OR (uganda[MeSH Terms])) OR (zambia[MeSH Terms])) OR (zimbabwe[MeSH Terms])) | 359 |
| Google Scholar | patient education OR educational therapy OR therapeutic education OR therapeutic patient education OR self-management education OR patient compliance OR self-management OR self-care OR programme AND diabetes OR diabetic AND Africa | 100 |
| CINAHL | TI Diabetes OR TI diabetic AND TI patient education OR AB patient education OR TI self-management OR AB self-management OR TI patient compliance OR AB patient compliance OR TI programme OR TI therapeutic education OR AB therapeutic education AND AB Africa. Geographical subset: Africa | 947 |
| Web of Science | ((((((((((ALL=(patient education)) OR ALL=(educational therapy)) OR ALL=(therapeutic education)) OR ALL=(therapeutic patient education)) OR ALL=(self-management education )) OR ALL=(patient compliance)) OR ALL=(self-management)) OR ALL=(self-care)) OR ALL=(intervention programme)) AND ALL=((diabetes)) OR ALL=(diabetes mellitus )) AND ALL=(Africa). Refined by diabetes. | 1091 |
