# Supplementary material for: Therapeutic patient education programs on diabetes in sub-Saharan Africa: A systematic review
Source: PLoS One. 2024 Jun 27;19(6):e0299526. doi: 10.1371/journal.pone.0299526 (PMC11210798; doi:10.1371/journal.pone.0299526)
Supplement: Attachment — Submitted filename: REVIEW REPORT.docx [file pone.0299526.s003.docx]

Thank you for the opportunity to review this study which seeks to explore the role of TPE programs in the health outcomes of individuals with diabetes in sub-Saharan Africa. The study is timely, given the limited data on the topic in the SSA region. It is well-written and interesting to read. I think it will add to and extend the existing knowledge on the topic in the literature. It is, thus, publishable.

Below are some suggestions which I think the authors may use to improve the overall quality of the paper:

1. Since the authors talk about diabetes in general, readers will benefit more if they include in the introduction a brief description of the types of diabetes: type 1, type 2, gestational diabetes, etc.
2. In lines 122 and 123, the authors state “No systematic review has been done on the evaluation of diabetes TPE programs in sub-Saharan Africa. This statement may not hold entirely. There is a similar review (Kumah E, Otchere G, Ankomah SE, Fusheini A, Kokuro C, Aduo-Adjei K, et al. Diabetes self-management education interventions in the WHO African Region: A scoping review. PLoS ONE 2021;16(8): e0256123. https://doi.org/10.1371/journal.pone.0256123) that has been conducted on the topic in the African Region. Although the authors did not specifically state that the study evaluates diabetes patient education interventions, they ended up evaluating the effectiveness of the included interventions. I would therefore suggest that the authors cite this study and state that the current study adds to and extends this existing study. And sub-Saharan Africa is not different from the WHO African region. Thus, some similarity exists between the current study and the previous study. On the other hand, if the authors see no semblance between their study and the existing one, they need to cite this existing study and indicate how it is different from the present study.
3. Search strategy: I would include at least one African health electronic database, such as African Index Medicus (AIM) in the electronic database searching since the study is focused on the SSA region.
4. Line 163, what do the authors mean by "uncontrolled studies" and how is this different from observational studies?
5. Figure 1: What do the authors mean by additional records identified through other sources? Since apart from the electronic database searching, they did not indicate additional searches done through other sources, which in my opinion could have been done by screening the reference lists of the eligible papers.
6. Results: Lines 184 and 185, further explanation is needed for excluding a mixed methods study from the review, because this may have a quantitative aspect that might meet the inclusion criteria. Sometimes, in mixed methods studies, qualitative aspects are added to explain the findings of the quantitative aspects. Thus, the excluded stud(ies) may have a diabetes education intervention, with a qualitative arm.
7. Instead of diabetic patients or patients with diabetes used throughout the study, could the authors use "individuals with diabetes" or "persons with diabetes" or "people with diabetes"
8. I feel Table 1 is overloaded. Could the authors break this down into two tables instead?
9. I would present the risk of bias assessment results before presenting the main findings of the study.

Thank you
